# Intermittent Pulse and Palpitation

**Published:** 1868-09

**Authors:** 


### EDITORIAL DEPARTMENT.

*Intermittent Pulse and Palpitation.*—Dr. Benjamin W. Richardson of England, to whom the Dental profession, as well as the Medical, is indebted for much that is useful, concludes a recent lecture on this subject with the following precepts:

"There is, of course, no known specific for intermittent heart, but whenever the symptoms of intermittency is present, there are certain general lines of treatment which should always be enforced by the physician.

"1. In the case of young children, when the intermittency is clear, however infrequent it may be, the utmost care should be taken to avoid every source of mental excitement. A child so circumstanced should, under no pretence and for no purpose, be oppressed with study. He should be subjected to no amusements which powerfully excite the mind; he should at no time be exhausted by physical fatigue; he should be well fed, warmly clothed from head to foot, and, above all things, should be allowed to have abundant sleep. Ten to twelve hour's sleep is not a whit too much. Moreover, such a child should never be put to sleep with stories which excite dreams or cause alarm.

"2. In adults equal care should be taken, and, above all things, attempts should be made to remove impressions derived from any untoward event. Change of scene in this way often proves of essential service, while a carefully regulated diet, abstinence from exhausting pleasures, and abstinence from exhausting labor, especially mental labor of one particular kind, should be encouraged. Good sleep is here again the most valuable of remedies. Eight hours of sleep out of the twenty-four are essential—nine are still better. Two special points of advice are of vast moment to such persons. It not unfrequently happens that, by accident or by direct information, they learn the fact that their pulse does intermit. Then they begin to feel their own pulse, and become charged with dread of sudden death. As the disorder is of itself cerebral, this watchfulness and fear increase the frequency of intermittency. With these patients, a word from the physician, timely and firmly spoken, is often the best prescription. You assure them on your experience that their malady is not of necessity fatal; you command them not to inquire after the symptom, and if you can succeed in persuading them to your views, which you may honestly try to do with all your influence, you will effect the most marked improvement in their condition. Again, it sometimes happens that patients, concious of the failure of the heart, resort to alcoholic stimulants as a means of relief. For a moment, by exalting the cerebral activity, they experience relief from the alcohol, but the depression that follows calls the more rapidly for a return to the supposed remedy, and a factitious benefit leads to a habit which excites structural changes, and of all things hastens death.

"3. In case of sudden intermittency, with symptoms of cerebral congestion, depletive measures are sound. A purgative is essential and blistering at the back of the neck is always useful. I have seen great advantage in these cases from abstraction of a moderate quantity of blood by the cupping glasses.

"4. In extreme forms of cardiac intermittency, while all the general rules laid down in Nos. 1 and 2 hold good, it becomes often imperatively necessary to subdue the cerebral excitement and to induce cerebral rest. For this latter purpose, opium is the sheet anchor. It must be given freely when it is given, and not too freely. Small and repeated doses of opium excite, depress and give no rest. A full dose, equivalent to a grain or even two grains, on the contrary, produce no excitement, but give sound sleep, and that quietude of cerebral circulation which is essential to secure satisfactory relief. I have sometimes, where there was much depression, combined full doses of opium with full doses of quinia, and with marked benefit.

"5. Concerning old people who suffer from what may be called chronic intermittency without oppressing symptoms, no special rule requires to be laid down. They are themselves usually too tired of the excitements of life to care for them, and if they are not, then the observance of the the general principles applicable to children and adults extend equally to them."

